# Comparative transcriptomics with self-organizing map reveals cryptic photosynthetic differences between two accessions of North American Lake cress

**DOI:** 10.1038/s41598-018-21646-w

**Published:** 2018-02-19

**Authors:** Hokuto Nakayama, Tomoaki Sakamoto, Yuki Okegawa, Kaori Kaminoyama, Manabu Fujie, Yasunori Ichihashi, Tetsuya Kurata, Ken Motohashi, Ihsan Al-Shehbaz, Neelima Sinha, Seisuke Kimura

**Affiliations:** 1 Department of Plant Biology, University of California Davis, One Shields Avenue, Davis, CA 95616 USA; 2 Department of Bioresource and Environmental Sciences, Kyoto Sangyo University, Kamigamo-Motoyama, Kita-Ku, Kyoto, 603–8555 Japan; 3 Plant Global Education Project, Graduate School of Biological Sciences, Nara Institute of Science and Technology, Nara, 630–0192 Japan; 4 Okinawa Institute of Science and Technology, 1919–1 Tancha, Onna-son, Okinawa, 904–0412 Japan; 5 RIKEN Center for Sustainable Resource Science, 1–7–22, Suehiro, Tsurumi, Yokohama, 230–0045 Japan; 6 JST, PRESTO, 4–1–8 Honcho, Kawaguchi, Saitama, 332–0012 Japan; 7 Center for Ecological Evolutionary Developmental Biology, Kyoto Sangyo University, Kamigamo-Motoyama, Kita-Ku, Kyoto, 603–8555 Japan; 8 Missouri Botanical Garden, P.O. Box 299, St. Louis, MO 63166–0299 USA; 9 Present Address: Faculty of Life Sciences, Kyoto Sangyo University, Motoyama, Kamigamo, Kita-Ku, Kyoto, 603–8555 Japan; 10 Present Address: Graduate School of Life Sciences, Tohoku University, 6–3 Aoba, Aramaki, Aoba-ku, Sendai, 890–8578 Japan

## Abstract

Because natural variation in wild species is likely the result of local adaptation, it provides a valuable resource for understanding plant-environmental interactions. *Rorippa aquatica* (Brassicaceae) is a semi-aquatic North American plant with morphological differences between several accessions, but little information available on any physiological differences. Here, we surveyed the transcriptomes of two *R*. *aquatica* accessions and identified cryptic physiological differences between them. We first reconstructed a *Rorippa* phylogeny to confirm relationships between the accessions. We performed large-scale RNA-seq and *de novo* assembly; the resulting 87,754 unigenes were then annotated via comparisons to different databases. Between-accession physiological variation was identified with transcriptomes from both accessions. Transcriptome data were analyzed with principal component analysis and self-organizing map. Results of analyses suggested that photosynthetic capability differs between the accessions. Indeed, physiological experiments revealed between-accession variation in electron transport rate and the redox state of the plastoquinone pool. These results indicated that one accession may have adapted to differences in temperature or length of the growing season.

## Introduction

Recent studies involving non-model plant species have provided knowledge unobtainable from using only model plants^1^. Many of these studies have described molecular mechanisms underlying interspecific differences in morphology, physiology, and ecology^2–4^. In addition to interspecific differences, natural genetic variation within a population of a single species is garnering increasing attention from researchers^5,6^. For instance, accessions of *Arabidopsis thaliana* (L.) Heynh. (hereafter “Arabidopsis”) vary in traits such as leaf morphology, flowering time, and drought response^6^, suggesting the effect of local adaptation. Several studies have addressed the evolutionary processes underlying this variation through identifying genes or miRNAs responsible for between-accession differences, prompting increased attention on accessions as experimental material^6^. Accessions are particularly powerful for studying non-model species that do not have the genetic resources (e.g., mutants) seen in model organisms. Additionally, accessions are useful for understanding how local adaptation processes may have sculpted morphological and physiological differences among populations.

*Rorippa* Scop. (Brassicaceae or Cruciferae) comprises 86 species^7^ distributed on all continents except Antarctica^8^. The within-genus diversity has resulted in considerable attention, with *R*. *aquatica* (Eaton) E.J.Palmer & Steyermark, *R*. *amphibia* (L.) Besser, and *R*. *sylvestris* (L.) Besser being particularly well studied^9^. *Rorippa aquatica*, also known as lake cress, is a semi-aquatic North American plant distributed east of the 95^th^ meridian from eastern Wisconsin into Quebec and southern Vermont into Florida^10,11^. This species is well adapted to the aquatic environment and exhibits heterophylly^12^, which is leaf-form variation on a single plant in response to surrounding environmental cues. In nature, deeply dissected leaves develop when plants grow in submerged conditions, whereas simple leaves with entire or toothed margins develop when grown on land^12^. Previously, we showed that *R*. *aquatica* leaf shape changes dramatically in response to varying ambient temperatures and submergence underwater^13^: an ambient temperature of 25 °C induced leaves with simpler forms compared with 20 °C. Additionally, we found that environmental variation (e.g., in ambient temperature and water levels) altered the expression levels of *KNOTTED1*-*LIKE HOMEOBOX* (*KNOX1*) orthologs; moreover, gibberellin accumulation, thought to be regulated by *KNOX1* genes, also changed in leaf primordia.

**Figure 1:**
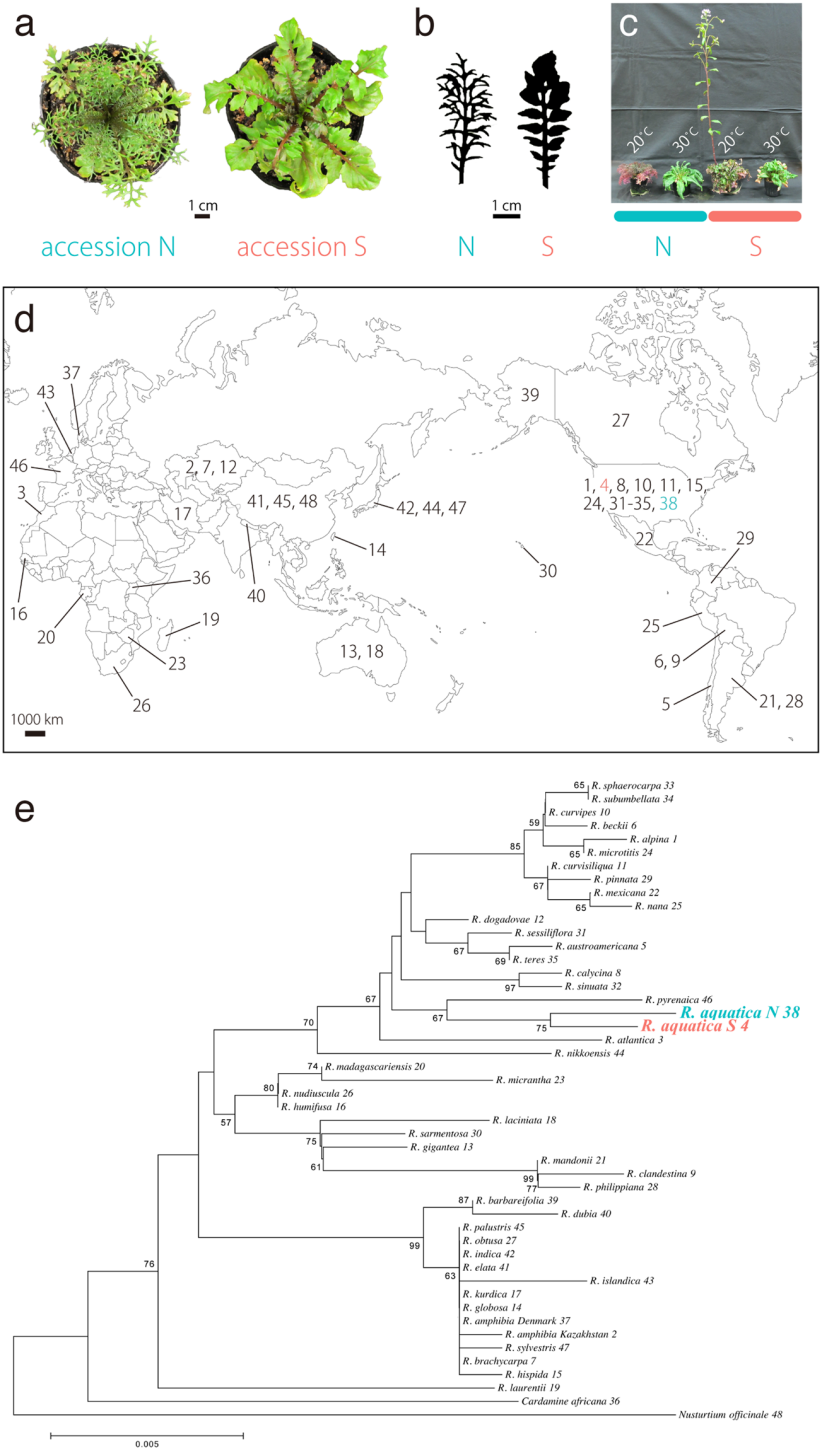
Comparison of leaf morphology in *Rorippa aquatica* accessions and Phylogenetic trees constructed using cpDNA sequences. (**a**) Top view of shoots in accession N (left) and S (right). Plants were cultivated in a growth chamber for a month at 20 °C and under continuous illumination (light intensity of 60 µmol photons m^−2^ s^−1^). (**b**) Comparison of morphology in accession N (left) and S (right). Plants were grown under the same conditions described in (a). (**c**) Comparison of flowering time between accessions. Side view of shoots in accession N and S. These plants were grown for three months under each listed condition (light intensity of 60 µmol photons m^−2^ s^−1^). (**d**) Global distribution of *Rorippa* species. Numbers within each country correspond to the species used in the phylogenetic analysis. The map was generated by using Illustrator CS4 (Adobe Systems). (**e**) Evolutionary history was inferred using the neighbor-joining method. The bootstrap values are indicated on branches (only those > 50% are indicated on the tree). The tree is drawn to scale, with branch lengths in the same units as the evolutionary distances used to infer the phylogeny.

*Rorippa aquatica* accessions^14^ from northern and southern United States clearly differed in leaf forms (Fig. 1a,b) under the same conditions. For instance, the northern sample (hereafter “accession N”) develops leaves with more complex forms than the southern sample (hereafter “accession S”). In addition to the morphological difference, accession N flowers later than accession S (Fig. 1c)^15^. In *Populus angustifolia*, it is known that northern and southern populations differ in photosynthetic physiology corresponding to latitude across the North American continent^16^. Therefore, there is a possibility that *Rorippa* accessions have a difference in photosynthetic activity. However, little is known about physiological differences between these accessions except for flowering time. Depending on environmental conditions, gene expression would be expected to vary across accessions, and these cryptic physiological differences can be uncovered with comparative transcriptome analysis using RNA-seq technology^17^.

In this study, we aimed to understand how local adaptation processes may have sculpted physiological differences between *R*. *aquatica* accessions. We performed large-scale RNA-seq, *de novo* assembly, and transcriptome annotation in addition to phylogeny reconstruction in *Rorippa*. Moreover, we variance-scaled transcriptome data separately by two accessions and compared them using principal component analysis (PCA) and self-organizing map (SOM) analysis. These methods provide more details on difference in expression pattern between accessions among different conditions than simple analyses of differential gene expression levels, because the scaling procedure allows focus on genes that exhibit between-accession variation in expression patterns. Then, based on SOM clustering results, we focused on genes with differential expression patterns between accessions. This comparative transcriptome analysis revealed cryptic differences between accessions, specifically in photosynthetic activity (e.g., electron transport rate) and the redox state of the plastoquinone pool.

## Results

### Accessions are closely related

Despite the attention paid to various *Rorippa* species, relatively little is known about their phylogenetic relationships. In particular, there was no report on phylogenetic relationship among *Rorippa* accessions. Sequences of cpDNA were determined from 46 samples of *Rorippa* species distributed worldwide and two samples from outgroups *Nasturtium officinale* W.T.Aiton and *Cardamine africana* L. (Fig. 1d; Table 1). In the NJ phylogenetic tree generated, all *Rorippa* samples (including *R*. *aquatica* accessions N and S) formed a monophyletic group, with the two accessions being the most closely related (Fig. 1e). These relationships were also confirmed in the ML phylogenetic tree (see Supplementary Fig. S1). The NJ phylogeny also suggested that *R*. *aquatica* is close to the European *R*. *pyrenaica* (L.) Reichenb., but the latter is not heterophyllous^18^. However, heterophylly is well documented in *R*. *amphibia*, a widespread Eurasian species naturalized in North America^19^. The latter species is placed in an entirely different clade from *R*. *aquatica* within *Rorippa* (Fig. 1e). Therefore, it seems to likely that heterophylly evolved independently at least twice within the genus.

**Table 1:** List of species, voucher numbers, and accession numbers of plant materials. Herbarium acronyms follow Index Herbariorum Part I.

ID #	Species	Locality	Voucher	*trnL* intron	*trnG*-*trnM*	*psbC*-*trnS*	Sampling
1	*R. alpina*	USA, Nevada	A. Tiehm, (MO)	LC194527	LC194528	LC194529	This study
2	*R. amphibia*	Kazakhstan	V. V. Byalt, (MO)	LC194530	LC194531	LC194532	This study
3	*R. atlantica*	Morocco	J. Gattefosse, (MO)	LC194533	LC194534	LC194535	This study
4	*R. aquatica* (S)	USA	Kyoto Sangyo Univ., (cult.)	LC194536	LC194537	LC194538	This study
5	*R. austroamericana*	Chile, Valparaiso	O. Zöllner (MO)	LC194539	LC194540	LC194541	This study
6	*R. beckii*	Bolivia	D.Collot, (MO)	LC194542	LC194543	LC194544	This study
7	*R. brachycarpa*	Kazakhstan	I. Al-Shehbaz, N. Aralbaev & S. Nesterova, (MO)	LC194545	LC194546	LC194547	This study
8	*R. calycina*	USA, Wyoming	R. Dorn, (MO)	LC194548	LC194549	LC194550	This study
9	*R. clandestina*	Bolivia, Santa Cruz	J. Abbott, (MO)	LC194551	LC194552	LC194553	This study
10	*R. curvipes*	USA, Utah	A. Kelsey and A. J. Moore, (MO)	LC194554	LC194555	LC194556	This study
11	*R. curvisiliqua*	USA, California	G. K. Helmkamp and E. A. Helmkamp, (MO)	LC194557	LC194558	LC194559	This study
12	*R. dogadovae*	Kazakhstan	A. Dogadova and N. Tzvelev, (MO)	LC194560	LC194561	LC194562	This study
13	*R. gigantea*	Australia, Queensland	W. J. McDonald, (MO)	LC194563	LC194564	LC194565	This study
14	*R. globosa*	China, Taiwan	C. M. Wang, (MO)	LC194566	LC194567	LC194568	This study
15	*R. hispida*	USA, Missouri	J. A. Steyermark, (MO)	LC194569	LC194570	LC194571	This study
16	*R. humifusa*	Senegal, Tambacounda	J. E. Madsen (MO)	LC194572	LC194573	LC194574	This study
17	*R. kurdica*	Iran	M. L. Grant, (MO)	LC194575	LC194576	LC194577	This study
18	*R. laciniata*	Australia, New South Wales	R. G. Coveny, (MO)	LC194578	LC194579	LC194580	This study
19	*R. laurentii*	Madagascar	H. Humbert, (MO)	LC194581	LC194582	LC194583	This study
20	*R. madagascariensis*	Gabon, Ogooué-Maritime	H. P. Bourobou *et al*., (MO)	LC194584	LC194585	LC194586	This study
21	*R. mandonii*	Argentina, Tucuman	M. Beilstein, (MO)	LC194587	LC194588	LC194589	This study
22	*R. mexicana*	Mexico, Durango	A. C. Sanders *et al*., (MO)	LC194590	LC194591	LC194592	This study
23	*R. micrantha*	Zimbabwe	J. F. Ngoni, (MO)	LC194593	LC194594	LC194595	This study
24	*R. microtitis*	USA, Arizona	J. Ricketson and V. Walter, (MO)	LC194596	LC194597	LC194598	This study
25	*R. nana*	Peru, Arequipa	W. Galiano, (MO)	LC194599	LC194600	LC194601	This study
26	*R. nudiuscula*	South Africa, Eastern Cape	V. R. Clark and S. Ramdhani, (MO)	LC194602	LC194603	LC194604	This study
27	*R. obtusa*	Canada, Ontario	C. F. Red, (MO)	LC194605	LC194606	LC194607	This study
28	*R. philippiana*	Argentina, San Juan	J. Chiapella and E. Vitek, (MO)	LC194608	LC194609	LC194610	This study
29	*R. pinnata*	Colombia, Cundinamarca	C. Parra-O. and J. L. Femandez-A., (MO)	LC194611	LC194612	LC194613	This study
30	*R. sarmentosa*	USA, Hawaii	G. Staples, (MO)	LC194614	LC194615	LC194616	This study
31	*R. sessiliflora*	USA, Missouri	T. E. Smith *et al*., (MO)	LC194617	LC194618	LC194619	This study
32	*R. sinuata*	USA, Missouri	B. Summers *et al*., (MO)	LC194620	LC194621	LC194622	This study
33	*R. sphaerocarpa*	USA, Arizona	J. S. Miller, (MO)	LC194623	LC194624	LC194625	This study
34	*R. subumbellata*	USA, California	G. L. Smith, (MO)	LC194626	LC194627	LC194628	This study
35	*R. teres*	USA, Florida	J. R. Abbott, (MO)	LC194629	LC194630	LC194631	This study
36	*C. africana*	Uganda	ATBP, (MO)	LC194632	LC194633	LC194634	This study
37	*R. amphibia*	Denmark, Jylland	A. Hansen, 198169, (TNS)	AB871924	AB871925	AB871926	Nakayama *et al*., 2014
38	*R. aquatica* (N)	USA	Kyoto Sangyo Univ., (cult.)	AB871891	AB871892	AB871893	Nakayama *et al*., 2014
39	*R. barbareifolia*	USA, Alaska	W. J. Cody & T. J. M. Webster, 5902, (TI)	AB871906	AB871907	AB871908	Nakayama *et al*., 2014
40	*R. dubia*	Nepal, Kathmandu	G. Murata *et al*., 6303314, (TI)	AB871912	AB871913	AB871914	Nakayama *et al*., 2014
41	*R. elata*	China, Baiyu Xian	D. E. Boufford *et al*., 37265, (TI)	AB871918	AB871919	AB871920	Nakayama *et al*., 2014
42	*R. indica*	Japn, Kyoto	Kyoto Sangyo Univ., (cult.)	AB871933	AB871934	AB871935	Nakayama *et al*., 2014
43	*R. islandica*	Netherlands, Sleeuwijk	A. C. de Roon, (TI)	AB871909	AB871910	AB871911	Nakayama *et al*., 2014
44	*R. nikkoensis*	Japan, Tochigi	J. Haginiwa, (TNS)	AB871927	AB871928	AB871929	Nakayama *et al*., 2014
45	*R. palustris*	China, Rangtang	D. E. Boufford *et al*., 39061, (TI)	AB871915	AB871916	AB871917	Nakayama *et al*., 2014
46	*R. pyrenaica*	France, Loire	F. Schltz, (TI)	AB871903	AB871904	AB871905	Nakayama *et al*., 2014
47	*R. sylvestris*	Japan, Fukui	S. Watanabe, 682661, (TNS)	AB871930	AB871931	AB871932	Nakayama *et al*., 2014
48	*N. officinale*	China, Derong Xian	D. E. Boufford *et al*., 30988, (TI)	AB871936	AB871937	AB871938	Nakayama *et al*., 2014

### Transcriptome sequencing, *de novo* assembly, and defining differentially expressed genes

*Rorippa aquatica* plants (two accessions, N and S) were planted in soil and grown at three temperatures (20 °C, 25 °C, 30 °C) in a growth chamber under continuous illumination, with a light intensity of 60 or 120 µmol photons m^−2^ s^−1^. Total RNA was extracted from the shoot apical meristem with subtending P1–P3 leaf primordia.

**Figure 2:**
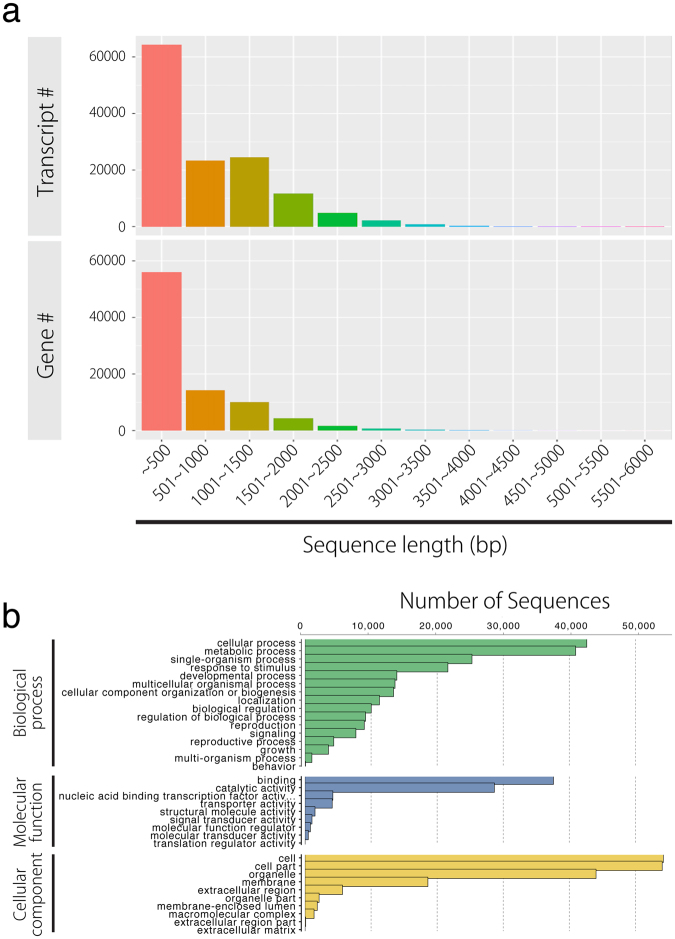
Transcripts, gene lengths, and gene ontology (GO) assignments for the *Rorippa aquatica* transcriptome. (**a**) Transcript and gene length distributions defined through *de novo* assembly in Trinity. (**b**) GO assignments predicting gene involvement. Top (green): biological processes; middle (blue): molecular function; bottom (yellow) cellular component. These assignments were generated in Blast2GO.

For *de novo* assembly, single-end sequencing of libraries with GAIIx (Illumina) resulted in 935,152,744 reads, and sequencing of longer reads was obtained through RNA-seq with MiSeq (Illumina) to yield 68,782,820 paired-end reads (Table 2). All reads from N and S were used for *de novo* assembly, because Trinity tries to generate a consensus transcript even if there is allelic variation. *De novo* assembly using all reads from N and S resulted in 132,566 transcript contigs, with N_50_ and average lengths of 1,031.06 nt and 1,903 nt, respectively (Table 2). Based on the N_50_ length, which is an indicator for assembly quality, we confirmed that the *de novo* assembly has enough quality. Approximately half of the transcripts were ≤500 nt (Fig. 2a; Table 2). Assembled sequences were annotated against the GO database. This procedure allows us to perform GO enrichment analysis, later. After annotation, the most predominant GO terms under the “biological process” category were as follows: cellular (GO: 0009987), metabolic (GO: 0008152), and single-organism (GO: 0044699), followed by response to stimulus (GO: 0050896) and developmental processes (GO: 0032502). Under “molecular function,” binding (GO: 0005488) and catalytic activity (GO: 0003824) were the most enriched terms. Under the “cellular component” category, cell (GO: 0005623), cell part (GO: 0044464), and organelle (GO: 0043226) were the most prominent (Fig. 2b). Reported RNA-seq data are available in the DDBJ Sequenced Read Archive under accession number DRA005242.

**Table 2:** Transcriptome sequencing and summary statistics of *de novo* assembly.

number/length	
Number of reads from GA IIx (32 bp; SE)	93,51,52,774
Number of reads from Miseq (2x300 bp; PE)	6,87,82,820
Total gene number	87,754
Total mRNA number	1,32,566
Ave. length of mRNA	1,031
Median	527
N_50_	1,903

For defining differentially expressed genes (DEGs) between accessions, we used only RNA-seq data from plants grown at 60 µmol photons m^−2^ s^−1^. Because, decreasing the number of environmental factors that similarly affect leaf form^13^, leaving only ambient temperature to vary. This reduced data complexity and facilitated further analysis. EdgeR was used to define 8,809 DEGs between the accessions (FDR < 0.01) based on a generalized linear model (GLM) at the gene level using temperature and accession as factors.

### Principal components analysis reveals differences in transcriptome profile between accessions

**Figure 3:**
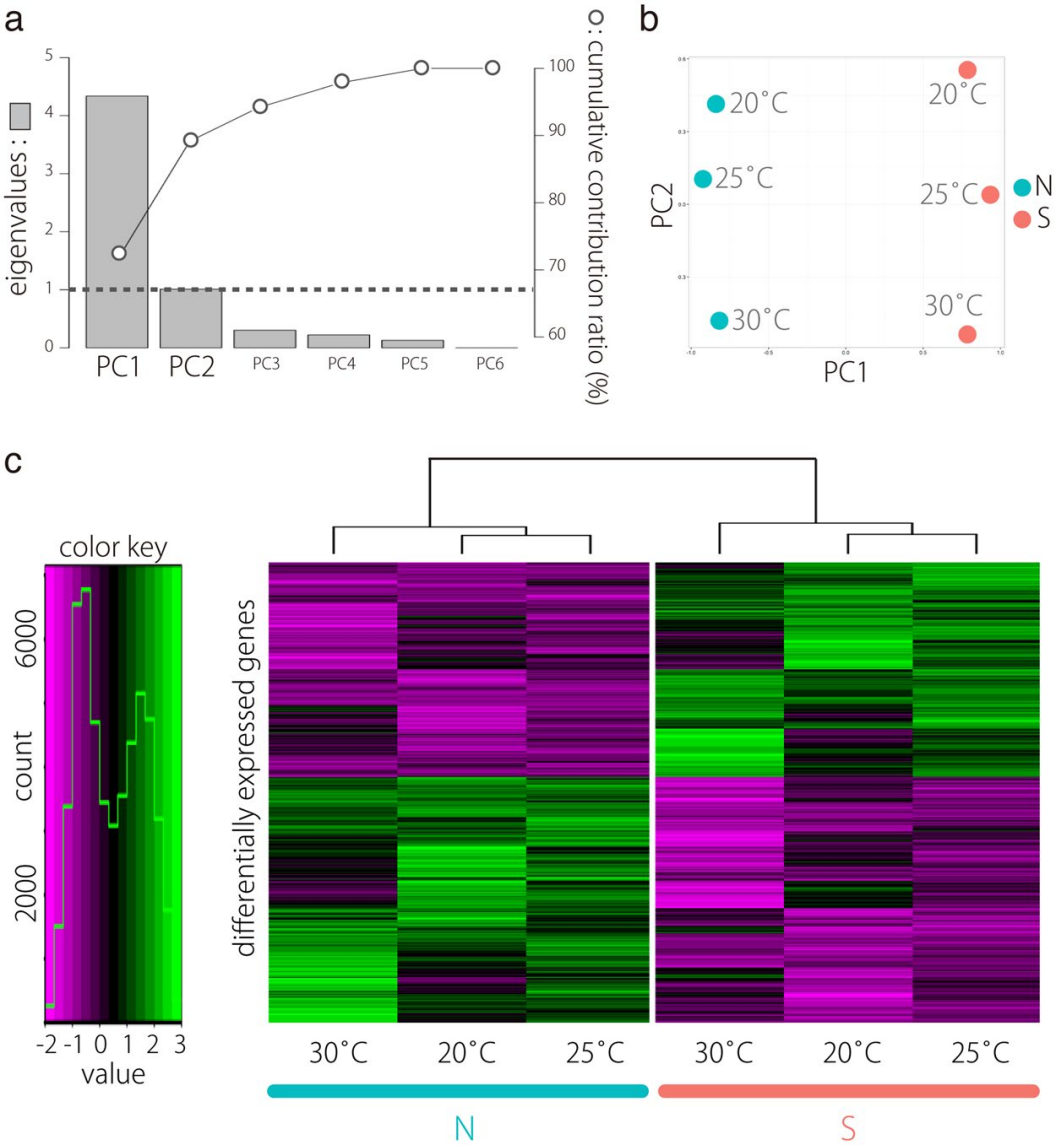
Principal component analysis (PCA) of gene expression. (**a**) Eigenvalues and cumulative contribution ratio (%) in PCA. Bars and open circles represent eigenvalues and cumulative contribution ratio, respectively. (**b**) The global expression profile of each transcript is represented as PC1 and PC2. Note distinct dissimilarities between the two accessions in PC1. (**c**) Expression profiles of genes that are differentially expressed between accessions.

To compare expression profiles between accessions, we performed PCA. Major sources of variance in the transcriptome were investigated with a PCA that considered all DEGs between accessions. The eigenvalues of two components were greater than 1 (Fig. 3a). The first component (PC1) explained 72.3% of the variation and discriminated clearly between accessions. The second component (PC2) explained 16.8% of the variation and discriminated between temperatures (Fig. 3a,b). Thus, the PCA results indicated that accessions differ in transcriptome profiles even under identical conditions. Indeed, a heatmap using all DEGs confirmed the PCA, showing clear differences in the expression patterns between accessions (Fig. 3c).

### Visualization and assessment of SOM clustering

We performed SOM for further understanding the difference in the expression patterns. SOM allows us to identify a subset of genes with similar expression profiles. We constructed a SOM to extract genes linked to between-accession physiological differences from DEGs between the accessions. We then used PCA to partition the resulting 20 SOM clusters following previous study^20^ (5 × 4, rectangular; Supplementary Fig. S2). The genes in each cluster exhibited distinct expression patterns along each condition, suggesting successful clustering (Supplementary Figs S2 and S3).

Expression patterns between accessions were similar in all clusters, differing mainly in degree even under the same conditions (Supplementary Figs S2 and S3). For instance, expression levels in cluster 10 decreased across both accessions as temperature increased, although the accessions differed in expression amount under identical temperatures. Therefore, it appears that each cluster contains genes showing different expression level and similar expression pattern between accessions. For further characterization of each cluster, we performed a GO enrichment analysis with the 20 clustered gene sets. “Response to stress” and “response to abiotic stress” GO terms were enriched in many clusters (q < 0.05), with the former being the top term in cluster 1 (see Supplementary Table S1). The strong representation of this term is likely a reflection of plant response to changes in ambient temperature, as expression levels of cluster 1 genes from both accessions increased with increasing temperature (Supplementary Figs S2 and S3). Moreover, the GO terms “post-embryonic development,” “multicellular organismal development,” “cell differentiation,” “anatomical structure morphogenesis,” and “cell growth” were enriched in cluster 10 (q < 0.05; see Supplemental Table S1). In this cluster, genes from accessions N and S decreased as temperature increased (Supplementary Figs S2 and S3), possibly reflecting a known relationship between temperature and leaf complexity^13^. These GO terms may be responsible for leaf-form differences across accessions, which exist even under the same environmental conditions (Fig. 1a,b). Furthermore, the “flower development” term was enriched in some clusters (q < 0.05), corresponding to between-accession differences in flowering time (Fig. 1c).

Overall, these results suggest that SOM clustering successfully identified distinct transcriptome differences between accessions. However, the large number of enriched GO terms prevented us from determining which gene types played a more critical role in influencing between-accession physiological differences.

### The use of SOM clustering on accession-scaled transcriptome data is sufficient for investigating cryptic differences between accessions

**Figure 4:**
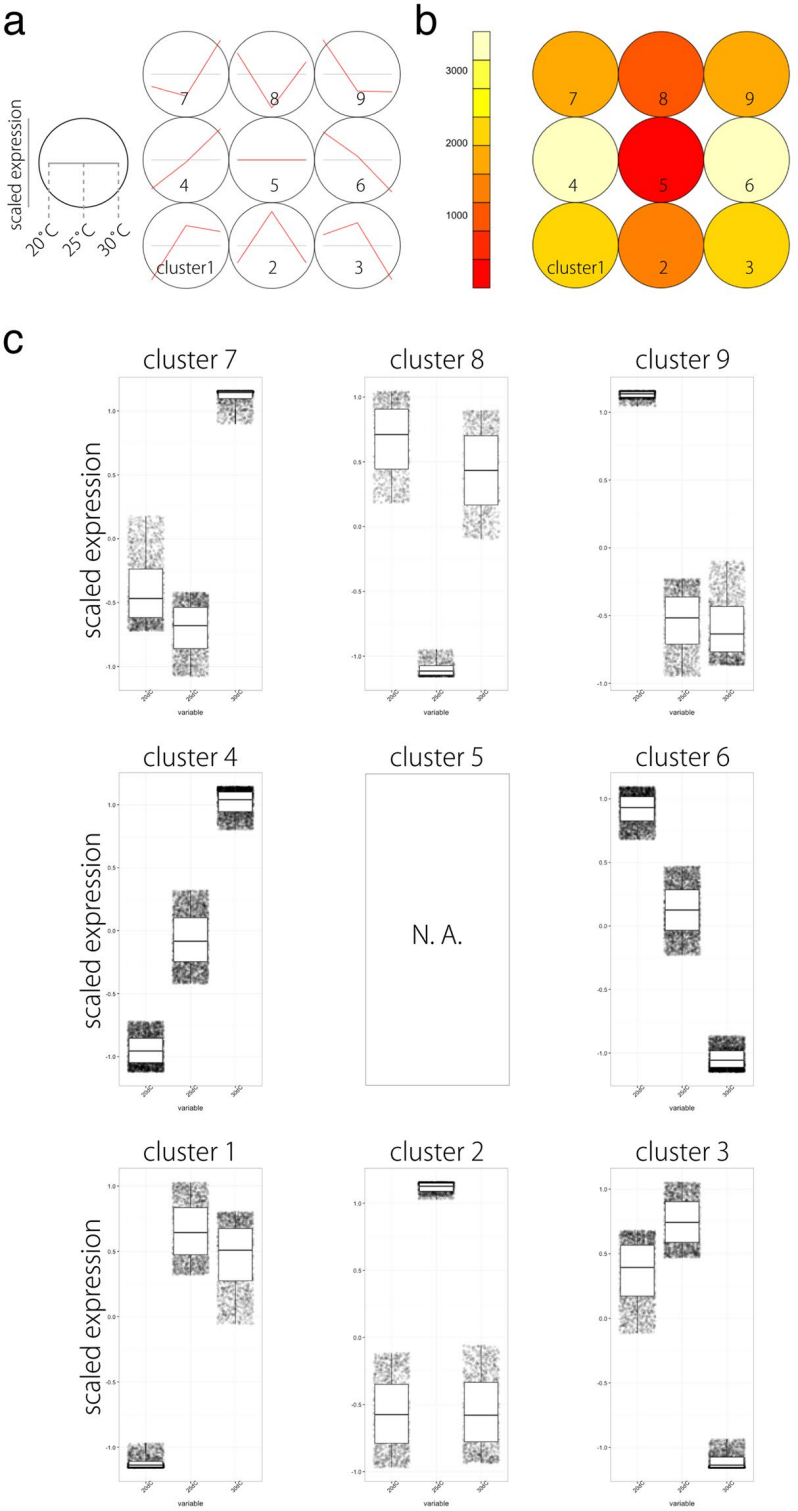
SOM clustering of gene expression in differentially expressed genes (DEGs) and their expression profiles. (**a**) Results of SOM clustering. Line plots indicate representative expression patterns at 20 °C, 25 °C, and 30 °C in each cluster. For SOM and diagrams, the 3 × 3 rectangular topology is shown. (**b**) Number of genes assigned to each SOM cluster. Red and white indicate low and high counts, respectively. (**c**) Scaled expression between accessions plotted under 20 °C, 25 °C, and 30 °C are shown. Box plot explanation: upper horizontal line of box, 75th percentile; lower horizontal line of box, 25th percentile; horizontal bar within box, median; upper horizontal bar outside box, 90th percentile; lower horizontal bar outside box, 10th percentile.

We next performed PCA and SOM clustering (3 × 3, rectangular) on count data of DEGs scaled separately by accession. Gene expression values from the accessions were mean-centered and variance-scaled separately to measure differences caused by changes in accession-specific expression patterns, allowing the focus to fall on differences in expression pattern instead of expression magnitude. Using such data allows separate treatment of genes from each accession and uncovers genes that cluster differently between accessions. As a result, genes from each accession were assigned to clusters irrespective of the accessions. Nine clusters were successfully obtained (Fig. 4a,b), based on box and line plots showing genes in each cluster with distinct, non-redundant expression patterns (Fig. 4c).

**Figure 5:**
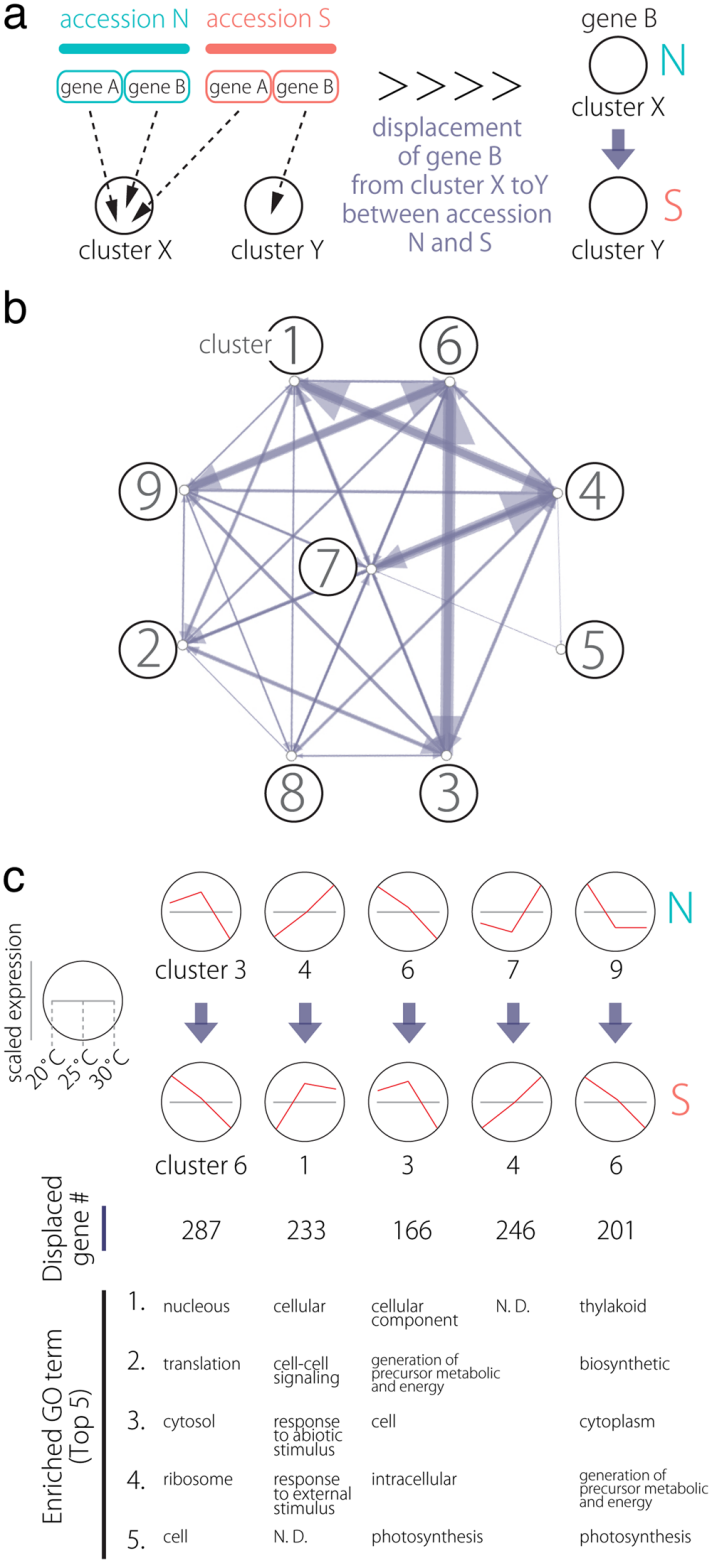
Displacement of orthologs to different clusters under the SOM clustering scheme. (**a**) A diagram demonstrating SOM clustering. N and S orthologs can be assigned to different clusters. (**b**) A network representation of ortholog assignment to different SOM clusters. Arrows represent displacement from accession N to S. Arrow sizes are proportional to the number of displaced orthologs. (**c**) Major displacement directions after SOM clustering of data that were scaled separately by accessions. Line plots indicate representative expression patterns in each cluster.

Next, we focused on genes with different between-accession expression patterns based on SOM clustering results (Fig. 5a). Such displaced gene sets between accessions among clusters exhibited certain tendencies (Fig. 5b; all directions from accession N to S). Pre- and post-displacement differences in expression pattern occurred primarily at 25 °C (Fig. 5c). GO enrichment analysis with these displaced gene sets between accessions among clusters showed that the GO term “photosynthesis” was significantly enriched in the displacements 3 → 6 (q value: 0.0346), 6 → 3 (0.0149), and 9 → 6 (0.000005), as were other photosynthesis-related GO terms, such as “thylakoid” (Table 3). Among the enriched genes were putative Arabidopsis orthologs of photosystem I subunit H-1 (AT3G16140), photosystem II subunit Q-2 (AT4G05180), *CURVATURE THYLAKOID 1 C* (AT1G52220), and NAD(P)H-quinone oxidoreductase subunit 2 A (ATCG00890) (see Supplementary Table S2). We confirmed that expression levels varied between accessions (see Supplementary Fig. S4). These results suggest that *R*. *aquatica* accessions differ physiologically in photosynthetic activity.

**Table 3:** Result of GO enrichment analysis using displacement of orthologs to different clusters under SOM clustering scheme.

cluster	GO term	adjusted P value by BH (q value)
3 → 6	nucleolus	6.42E-16
	translation	6.10E-14
	cytosol	1.32E-13
	ribosome	2.05E-12
	cell	2.04E-11
	structural molecule activity	2.04E-11
	cellular_component	2.63E-11
	protein metabolic process	1.80E-10
	intracellular	5.17E-09
	plastid	1.67E-07
	external encapsulating structure	1.69E-07
	cell wall	2.94E-07
	vacuole	5.25E-07
	cytoplasm	2.34E-06
	membrane	2.46E-05
	cellular component organization	8.04E-05
	cellular process	9.28E-05
	biosynthetic process	1.38E-04
	nucleus	3.05E-04
	nucleobase, nucleoside, nucleotide and nucleic acid metabolic process	2.52E-03
	generation of precursor metabolites and energy	3.06E-03
	plasma membrane	4.22E-03
	metabolic process	1.18E-02
	protein modification process	1.22E-02
	Golgi apparatus	1.34E-02
	biological_process	2.99E-02
	photosynthesis	3.46E-02
4 → 1	cellular_component	1.03E-02
	cell-cell signaling	4.40E-02
	response to abiotic stimulus	4.40E-02
	response to external stimulus	4.40E-02
6 → 3	cellular_component	6.82E-04
	generation of precursor metabolites and energy	1.27E-03
	cell	5.96E-03
	intracellular	1.46E-02
	photosynthesis	1.49E-02
	thylakoid	1.55E-02
	cellular component organization	3.18E-02
9 → 6	thylakoid	4.12E-07
	biosynthetic process	5.05E-06
	cytoplasm	5.05E-06
	generation of precursor metabolites and energy	5.05E-06
	photosynthesis	5.05E-06
	plastid	1.61E-05
	metabolic process	2.86E-05
	intracellular	5.23E-05
	cytosol	6.85E-05
	cell	8.55E-05
	carbohydrate metabolic process	1.06E-04
	cellular process	1.02E-03
	biological_process	1.04E-03
	membrane	1.64E-03
	cellular_component	2.14E-03
	catabolic process	5.18E-03
	cellular component organization	1.78E-02
	nucleobase, nucleoside, nucleotide and nucleic acid metabolic process	1.82E-02
	mitochondrion	2.59E-02
	secondary metabolic process	4.18E-02
	protein metabolic process	4.21E-02
	nucleotide binding	4.63E-02
	endosome	4.63E-02

**Figure 6:**
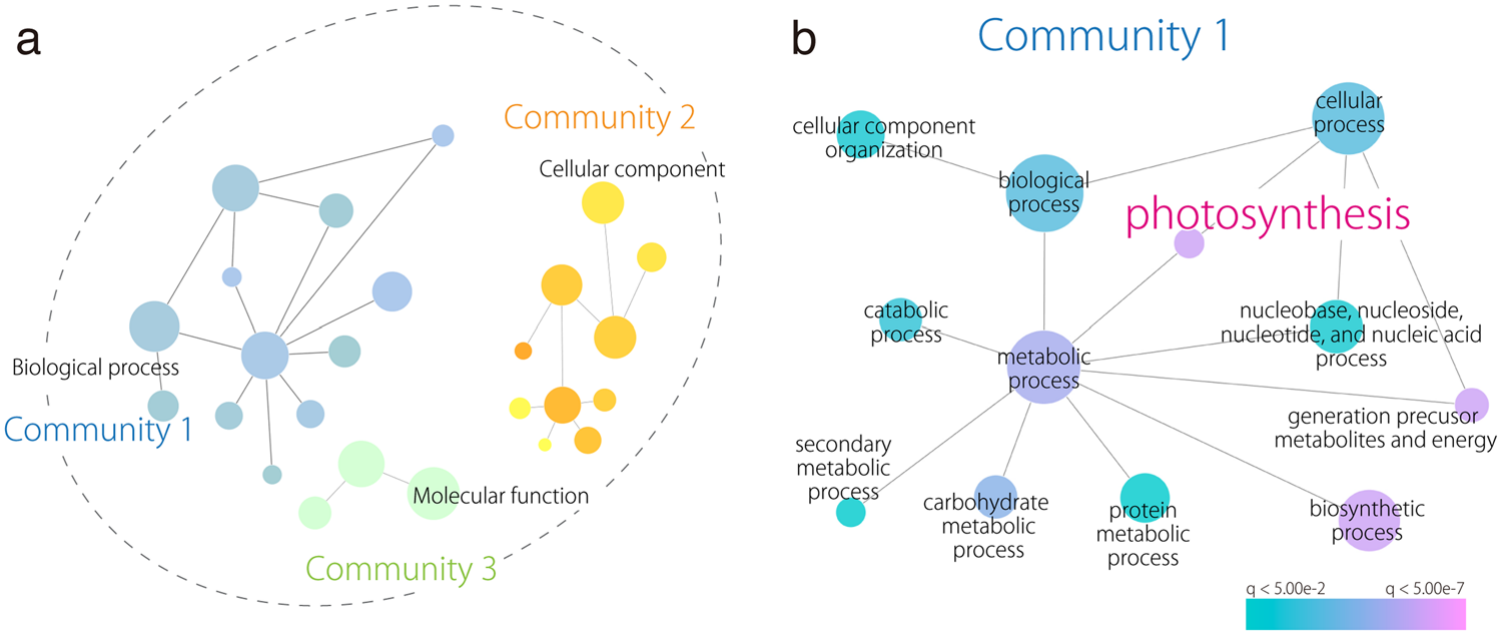
GO enrichment map with differentially expressed genes (DEGs) displaced from cluster 9 to cluster 6. (**a**) Three distinct communities (generated by Cytoscape) are on the map. (**b**) GO enrichment map of community 1 from (**a**). The red to blue scale indicates high to low q values, or P values adjusted with Benjamini-Hochberg.

As the q value of “photosynthesis” was the lowest in 9 → 6 compared with other displacements such as 3 → 6 and 6 → 3 (Table 3), we then constructed an enrichment map focused on GO terms in 9 → 6. The results showed that communities 1, 2, and 3 were represented by “Biological process,” “Cellular component,” and “Molecular function,” respectively (Fig. 6a). Community 2 comprised the enrichment of terms such as “thylakoid” and “cytoplasm.” In community 3, “nucleotide binding” was enriched (see Supplementary Fig. S5). In contrast, “photosynthesis” was significantly enriched under the “metabolic process” and “cellular process” GO terms in community 1 (Fig. 6b). Therefore, we investigated photosynthetic activity to verify the presence of between-accession differences.

### Electron transport rate (ETR) and redox state of the plastoquinone (PQ) pool are different between accessions

**Figure 7:**
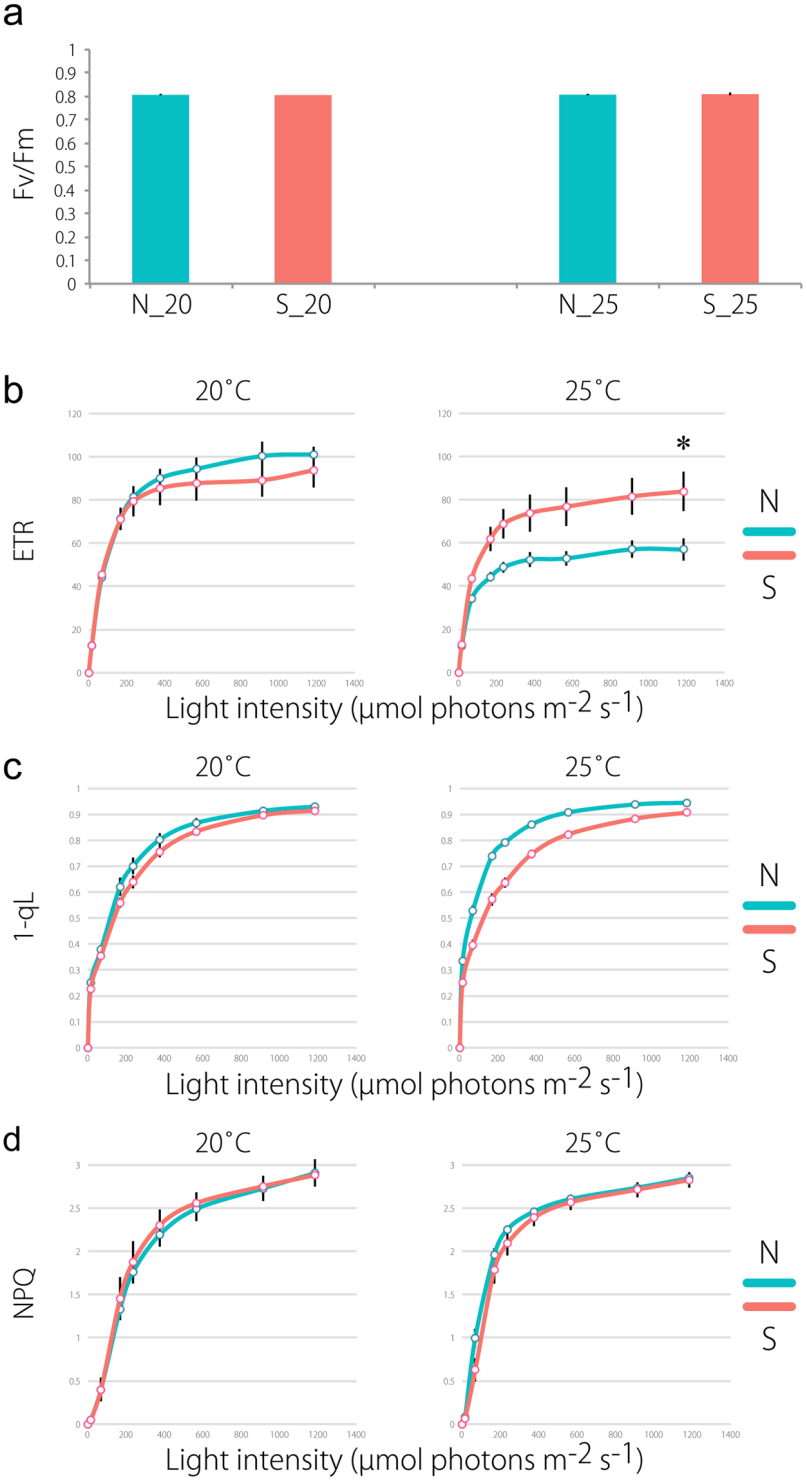
Measurements of photosynthetic parameters in two accessions. (**a**) Maximum quantum efficiency of photosystem II (Fv/Fm). (**b**) Light-intensity dependence of the electron transport rate (ETR). The ETR was calculated as Φ_PSII_ × light intensity (μmol photons m^−2^ s^−1^). (**c**) Light-intensity dependence of the redox state of plastoquinone (1-qL). (**d**) Light-intensity dependence of the non-photochemical quenching (NPQ) of chlorophyll fluorescence. All data are the means of five replicates; vertical bars represent SE. *p < 0.05 based on Welch’s t-tests.

Chlorophyll fluorescence parameters were analyzed to evaluate photosynthetic activity. In accessions N and S grown at 20 °C and 25 °C, PSII activity was high, with a maximum quantum yield (Fv/Fm) greater than 0.8 (Fig. 7a), indicating that photoinhibition was not observed. Under all light intensities, both accessions grown at 20 °C showed similar ETR (Fig. 7b), an indicator of the relative electron flow rate through PSII during steady-state photosynthesis. In contrast, accession N’s ETR values were lower than accession S at 25 °C and were saturated at a lower light intensity (Fig. 7b). To analyze electron transport in more detail, the 1-qL parameter, which reflects the redox state of the PQ pool, was measured. When grown at 25 °C, accession N had higher 1-qL than accession S, indicating a more electron-reduced PQ pool in the former (Fig. 7c). These results indicated that accession S had higher photosynthetic activity than accession N at 25 °C, but not at 20 °C. This is unsurprising because pre- and post-displacement differences in expression pattern occurred primarily at 25 °C (Fig. 5C). Additionally, we measured NPQ and observed no difference in NPQ induction between accessions (Fig. 7d).

Together, our data showed that between-accession differences in the expression of photosynthesis-related genes might contribute to the more active photosynthetic electron transfer system in accession S at warmer temperatures.

## Discussion

To investigate physiological differences between two *R*. *aquatica* accessions, we used phylogenetic, transcriptomic, bioinformatic, and physiological approaches. First, we reconstructed a phylogeny of *Rorippa* to confirm the relationship between two accessions with different habitats. Next, we performed large-scale RNA-seq, *de novo* assembly, and transcriptome annotation of the two accessions. We then compared these transcriptomes using PCA and SOM construction. We focused especially on genes with different between-accession expression patterns, based on comparisons of results from SOM clustering (Supplementary Fig. S6). The results suggested that photosynthetic capability, as measured by ETR and 1-qL, differs between the accessions. This difference may be an adaptive response to variation in growing season length or temperature. Overall, this study demonstrated that combining RNA-seq and clustering methods can reveal cryptic physiological differences between closely related accessions.

Previous studies showed that clustering methods combining PCA and SOM are effective in extracting gene subsets associated with phenotypes of interest from large-scale transcriptome data between species^20^. Although the use of PCA and SOM on transcriptome data identified numerous enriched GO terms related to between-accession physiological differences (including in photosynthesis), the sheer number of terms hampered our ability to focus on the most likely candidates. The high-dimensional data obtained from large-scale RNA-seq often requires simplification and conversion to become more interpretable^21^. Therefore, we reduced data dimensionality via scaling data separately by accessions before performing another PCA and SOM clustering. This fine-tuning let us uncover enrichment of photosynthesis-related genes (GO: 0015979; Q q value: 0.000005) in gene sets displaced between accessions among clusters. Indeed, our investigation of chlorophyll fluorescence parameters demonstrated between-accession differences in ETR and 1-qL, supporting results from the GO enrichment analysis. These results indicate that RNA-seq combined with SOM is remarkably effective for investigating cryptic differences between accessions, as long as data dimensionality is reduced first.

Physiological experiments revealed that accession S has higher ETR and lower 1-qL than accession N when both were grown at 25 °C, indicating that photosynthetic activity may be higher in accession S. When grown at 20 °C, however, accessions did not differ in their chlorophyll fluorescence parameters. Therefore, accession S may have a higher carbon fixation rate than accession N at 25 °C. Thus, these data suggest that accession S may be better adapted to 25 °C or higher temperatures.

The greater photosynthetic activity in accession S compared with accession N, particularly at higher temperatures, is useful for understanding the history of these two populations. The habitats of accessions S and N are thought to be respectively southern (e.g., Florida) and northern (e.g., Ohio and New England) United States^15^, spanning a wide range of temperatures and day lengths. These considerable environmental gradients can lead to local adaptation. It seems that our physiological experiment on photosynthetic activity provided evidence that accession S was better adapted to 25 °C than accession N. Indeed, the annual average temperature is 22 °C–26 °C in Florida and lower in Ohio (http://www.cpc.ncep.noaa.gov: National Weather Service Climate Prediction Center). Similarly, northern and southern *Populus angustifolia* populations differ in photosynthetic physiology corresponding to latitude across the North American continent; this variation may be an adaptive response to differences in growth season length, temperature, and insulation^16^. This relationship between photosynthetic physiology and latitude has also been reported in other North American plant species^22^. Thus, observed patterns in photosynthetic activity among *R*. *aquatica* accessions may be explained by similar adaptive measures.

Our method of combining RNA-seq and SOM was successful in detecting cryptic physiological differences between *R*. *aquatica* accessions. By using this method, further work could considerably clarify the molecular mechanisms underlying heterophylly in this species. Beyond *R*. *aquatica* research, this comparative technique has broad applications that can be improved further with recent advances in software, packages, and methods for fine-tuned transcriptome analysis^23–25^. Some of these analyses include predicting co-expression networks and defining participating modules, as well as investigating differential co-expression across disparate datasets. Indeed, this comparative transcriptome method has resulted in a gene network module regulating interspecific diversity in the genus *Solanum*^26^. Thus, comparative transcriptomics will contribute largely to uncovering key regulatory mechanisms affecting variation between and within species. The knowledge obtained from comparative transcriptomics will provide fundamental insight into evolutionary and ecological developmental biology, especially on the concept of rewiring network interactions during evolution, a process that can lead to speciation and local adaptation.

## Methods

### Plant materials

*Rorippa aquatica* plants (two accessions, N and S) were planted in soil and grown at three temperatures (20 °C, 25 °C, 30 °C) in a growth chamber under continuous illumination, with a light intensity of 60 or 120 µmol photons m^−2^ s^−1^. Seedlings were watered every two days. According to previous reports, N and S accessions are thought to have representative phenotypes from northern and southern populations^10,11,15^. All plants were cultivated in each condition for a month except those used for the physiological experiment, which were cultivated for two months. The shoot apical meristem subtending P1–P3 leaf primordia were frozen in liquid nitrogen just after sampling, and then stored at −80 °C until needed for DNA and RNA extraction.

### Phylogenetic analyses

Phylogenetic trees were reconstructed in MEGA6^27^ with the neighbor-joining (NJ) and maximum-likelihood (ML) methods^28,29^. Bootstrap values were derived from 1000 replicate runs.

Sequences of the non-coding regions in the *trnL* intron, *trnG* (GCC)-*trnM* (CAU), and *psbC*-*trnS* (UGA) were determined from 46 samples of *Rorippa* species distributed worldwide and two samples from outgroups *Nasturtium officinale* W.T.Aiton and *Cardamine africana* L. (Table 1). All sequence data were deposited in the DNA Data Bank of Japan (DDBJ) (Table 1). Their lengths were 517–527 bp for *trnL* intron, 224–228 bp for *trnG*-*trnM*, and 205–222 bp for *psbC*-*trnS*.

The optimal NJ phylogenetic tree is shown in Fig. 1e (sum of branch lengths = 0.14081464), along with relationships between the clades and localities of individuals (see also Table 1). A bootstrap test of 1000 replicates^30^ was used to calculate the percentage of replicate trees in which the associated taxa clustered together.

Evolutionary distances (number of base substitutions per site) were computed using maximum composite likelihood (MCL). The analysis involved 48 nucleotide sequences. Included codon positions were 1st + 2nd + 3rd + Noncoding, while all positions containing gaps and missing data were eliminated, resulting in a final dataset of 910 positions.

The ML phylogenetic tree with the highest log likelihood (-2191.1860) is shown in Supplemental Fig. S1. Initial tree(s) for the heuristic search were obtained automatically: Neighbor-Join and BioNJ algorithms were applied to a matrix of pairwise distances estimated with MCL, and then the topology with a superior log likelihood value was selected. The tree is drawn to scale, with branch lengths measured in the number of substitutions per site. The analysis involved 48 nucleotide sequences. Codon positions included were 1st + 2nd + 3rd + Noncoding. All positions containing gaps and missing data were eliminated to result in a final dataset of 910 positions.

### RNA-seq and *de novo* assembly

Total RNA was extracted from the shoot apical meristem with subtending P1–P3 leaf primordia and shoot with an RNeasy Plant Mini Kit (QIAGEN), for multiplex sequencing in the Illumina Genome Analyzer IIx (Illumina). RNA-seq libraries were prepared using a NEBNext mRNA Library Prep Reagent Set for Illumina (NEB). To find differentially expressed genes (DEGs), 48 libraries (two accessions, three temperatures, two light intensities, and four biological replicates) were prepared. *De novo* assembly was generated with RNA from several controlled growth conditions (see “Plant materials”), because changes in ambient temperature and light intensity affect leaf morphology^13^, and because certain transcripts may only be expressed in specific environments.

Longer reads for *de novo* assembly were obtained through RNA-seq with MiSeq (Illumina). Total RNA was extracted from the shoot apex subtending the leaf primordia. Libraries for MiSeq were prepared with a TruSeq Stranded Total RNA Sample Prep Guide (Illumina), and sequenced with a MiSeq Reagent Kit v3, both following manufacturer protocols.

Short single-end and long paired-end reads were assembled into transcriptome contigs using Trinity^31^, with default assembling settings. The minimum assembled contig length in our study is 200 bp. BlastX searches of obtained contigs against non-redundant protein sequences from GenPept, SwissProt, PIR, PDF, PDB, and NCBI RefSeq (nr) databases were conducted to find similar known protein sequences. Gene ontology (GO) information was mapped to each contig based on Blastx results with Blast2GO^32^.

### Gene expression profiling with RNA-seq data

Single-end reads were separated by indices, then trimmed and quality-filtered. Raw reads were then mapped with BWA^33^ (http://bio-bwa.sourceforge.net). Contigs from *de novo* assembly were used as reference sequences for mapping. Transcript expression profiles and DEGs were defined with EdgeR GLMs^34^. After quality filtering, 93.4% (80,304,302) of the single-end reads were mapped to the reference *de novo* assembly data using BWA version 0.7.5 (parameters “-n 2 -e 2”). For further analysis in R (version 3.2.1), lowly expressed genes were filtered based on a minimum sum of 10 counts over all samples (genes below this threshold were considered not expressed). Libraries were subjected to trimmed mean of M-values (TMM) normalization in EdgeR. Multi-dimensional scaling was performed via calculating log-fold changes between accessions and using DEGs to compute distances in EdgeR with the “plotMDS” function. Differential expression was calculated via fitting a generalized linear model (GLM) at the gene level using temperature and accession as factors. The threshold for DEGs was a false discovery rate (FDR) of < 0.01; this yielded 8,809 genes. Bioinformatics and statistical analyses were performed on the iPLANT Atmosphere cloud server (http://www.iplantcollaborative.org).

### Principal components analysis with SOM clustering and GO analysis

We applied a gene-expression clustering method^20^ on all 8,809 DEGs defined with EdgeR. Scaled expression values were used for multilevel 5 × 4 and 3 × 3 rectangular SOM clusters (Supplementary Fig. S6)^35,36^. One hundred training interactions were used during clustering, and gene clusters were based on the final assignment of genes to winning units. To focus only on gene-expression patterns instead of expression magnitude, expression values were mean-centered and variance-scaled separately between accessions in a 3 × 3 rectangular SOM. Using such data allows separate treatment of genes from each accession and uncovers orthologs that cluster differently based on their existing groups (e.g., accessions or species^20^). This procedure makes it possible to focus on genes that vary in expression patterns between accessions.

The outcome was then visualized in a PCA, with PC values calculated from gene expression across samples (R stats package, prcomp function). For 3 × 3 rectangular SOM clusters, network graphics in Gephi^37^ were used to visualize—as a directed network—the assignment of genes from different accessions to separate clusters. Arrow direction indicates gene assignment to clusters, from accession N to accession S, with arrow size proportional to gene number represented. Clustered and displaced gene sets among clusters were then subjected to GO analysis using Cytoscape and visualized with the BinGO^38^ (http://apps.cytoscape.org/apps/bingo). Resultant P values were adjusted with the Benjamini-Hochberg method to yield q values. Blast2GO results were used as annotation data.

### Chlorophyll fluorescence analysis

Chlorophyll fluorescence was measured with a Mini-PAM (pulse-amplitude modulation) portable chlorophyll fluorometer (Walz). For this analysis, all plants were grown under each environmental condition for two months. Minimum fluorescence (Fo) was obtained with open Photosystem II (PSII) centers in the dark-adapted state through a low-intensity measuring light (wavelength 650 nm, 0.05–0.1 μmol photons m^−2^ s^−1^). A saturating pulse of white light was applied to determine the maximum fluorescence with closed PSII centers in the dark-adapted state (Fm) and during actinic light (AL) illumination (Fm′). The steady-state fluorescence level (Fs) was recorded during AL illumination (17–1184 μmol photons m^−2^ s^−1^). The quantum yield of PSII (Φ_PSII_) was calculated as (Fm′ − Fs)/Fm^39^. The relative rate of electron transport through PSII (ETR) was calculated as Φ_PSII_ × light intensity (μmol photons m^−2^ s^−1^). The fraction of the open PSII center (qL) was calculated as [Φ_PSII_/(1 – Φ_PSII_)] × [(1 − Fv/Fm)/(Fv/Fm)] × (NPQ + 1)^40^. Non-photochemical quenching (NPQ) was calculated as (Fm − Fm′)/Fm′; this parameter is roughly indicative of excess absorbed light dissipation as heat to minimize oxygen radical formation in angiosperms. To analyze light-intensity dependence of fluorescence parameters, AL intensity was increased in a step-wise manner every two minutes after applying a saturating pulse.

## Electronic supplementary material

Supplementary Information

Supplementary Table1
