# Supplementary material for: Comparative transcriptomics with self-organizing map reveals cryptic photosynthetic differences between two accessions of North American Lake cress
Source: Sci Rep. 2018 Feb 19;8:3302. doi: 10.1038/s41598-018-21646-w (PMC5818620; doi:10.1038/s41598-018-21646-w)
Supplement: Supplementary file 1 — Supplementary Information [file 41598_2018_21646_MOESM1_ESM.pdf]

Supplementary Information

Comparative transcriptomics with self-organizing map reveals cryptic photosynthetic differences between two accessions of North American Lake cress

[1,2+]Hokuto Nakayama, [3,a+]Tomoaki Sakamoto, [2]Yuki Okegawa, [2]Kaori Kaminoyama, [4]Manabu Fujie, [5,6]Yasunori Ichihashi, [3,b]Tetsuya Kurata, [2,7]Ken Motohashi, [8]Ihsan Al-Shehbaz, [1]Neelima Sinha, and [2,7*]Seisuke Kimura

[1] Department of Plant Biology, University of California Davis, One Shields Avenue, Davis, CA 95616, U.S.A.

[2] Department of Bioresource and Environmental Sciences, Kyoto Sangyo University, Kamigamo-Motoyama, Kita-Ku, Kyoto 603-8555, Japan

[3] Plant Global Education Project, Graduate School of Biological Sciences, Nara Institute of Science and Technology, Nara 630-0192, Japan

[4] Okinawa Institute of Science and Technology, 1919-1 Tancha, Onna-son, Okinawa 904-0412, Japan

[5] RIKEN Center for Sustainable Resource Science, 1-7-22, Suehiro, Tsurumi, Yokohama 230-0045, Japan

[6] JST, PRESTO, 4-1-8 Honcho, Kawaguchi, Saitama 332-0012, Japan

[7] Center for Ecological Evolutionary Developmental Biology, Kyoto Sangyo University, Kamigamo-Motoyama, Kita-Ku, Kyoto 603-8555, Japan

[8] Missouri Botanical Garden, P.O. Box 299, St. Louis, MO 63166-0299, U.S.A.

[*]seisuke@cc.kyoto-su.ac.jp

[+]These authors contributed equally to this work.

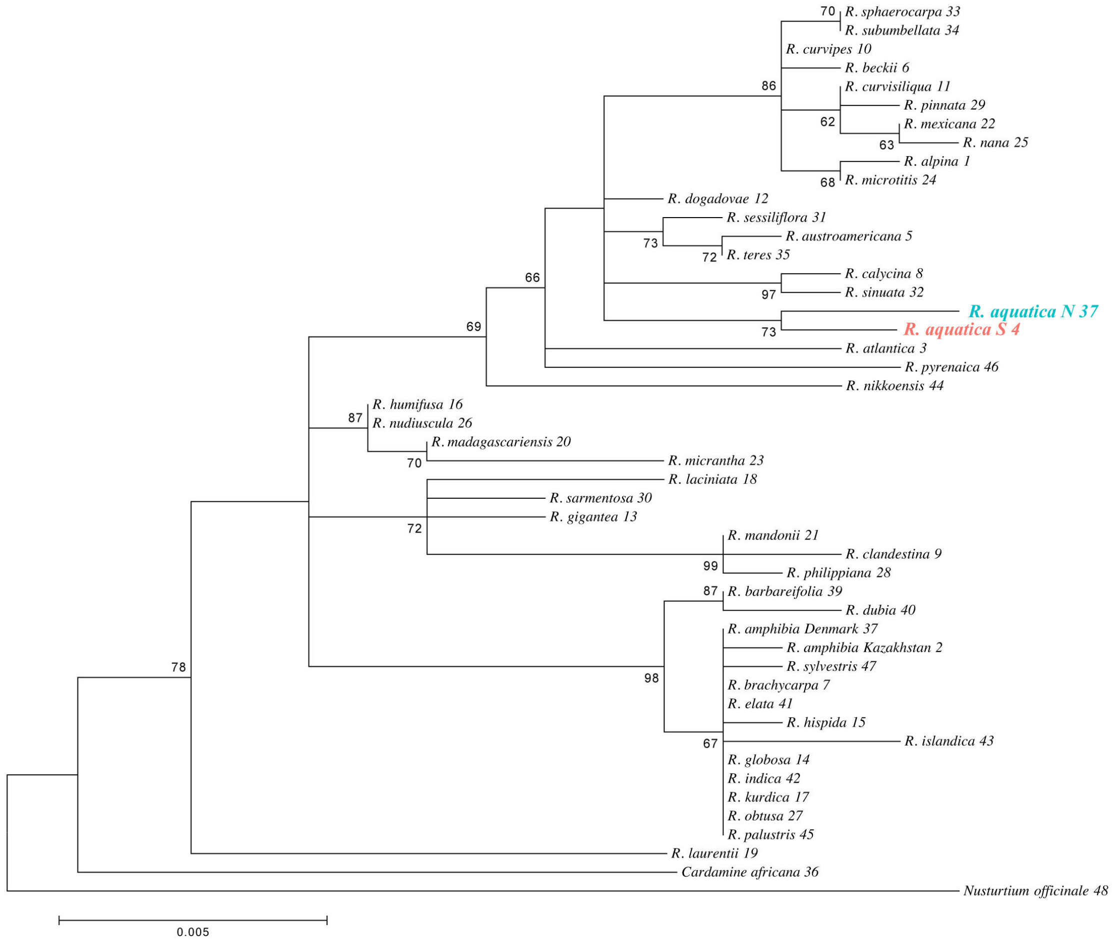

Supplementary Figure S1. Molecular phylogenetic analysis of cpDNA sequences using maximum likelihood.

The numbers next to nodes indicate percentage of trees (>50 %) in which associated taxa clustered together. The tree is drawn to scale, with branch lengths measured in the number of substitutions per site.

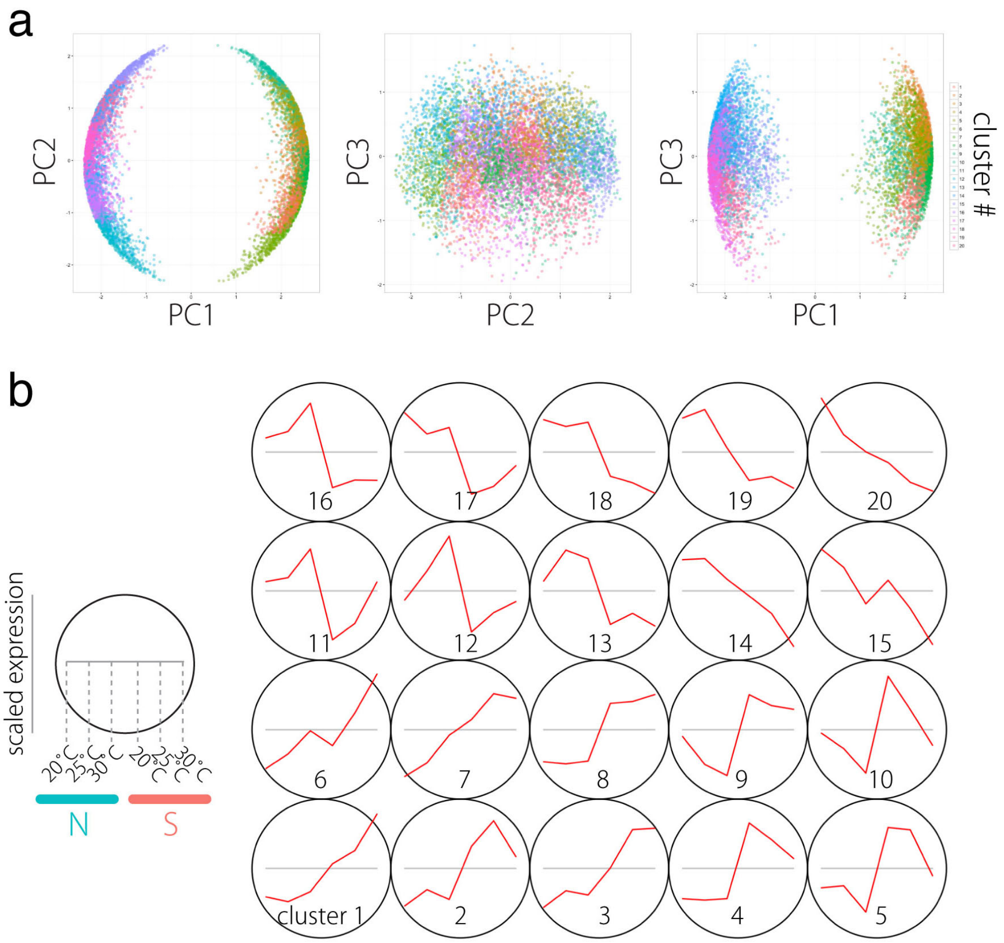

Supplementary Figure S2. Clustering of gene expression in differentially expressed genes (DEGs).

(a) PCA was performed on DEGs in the two accessions. Genes belonging to different self-organizing map (SOM) clusters are indicated with different colors and projected as PC1 and PC2. Color assignments are arbitrary. (b) Results of SOM clustering. Line plots indicate representative expression patterns in each cluster. For SOM and diagrams, the 5 × 4 rectangular topology is shown.

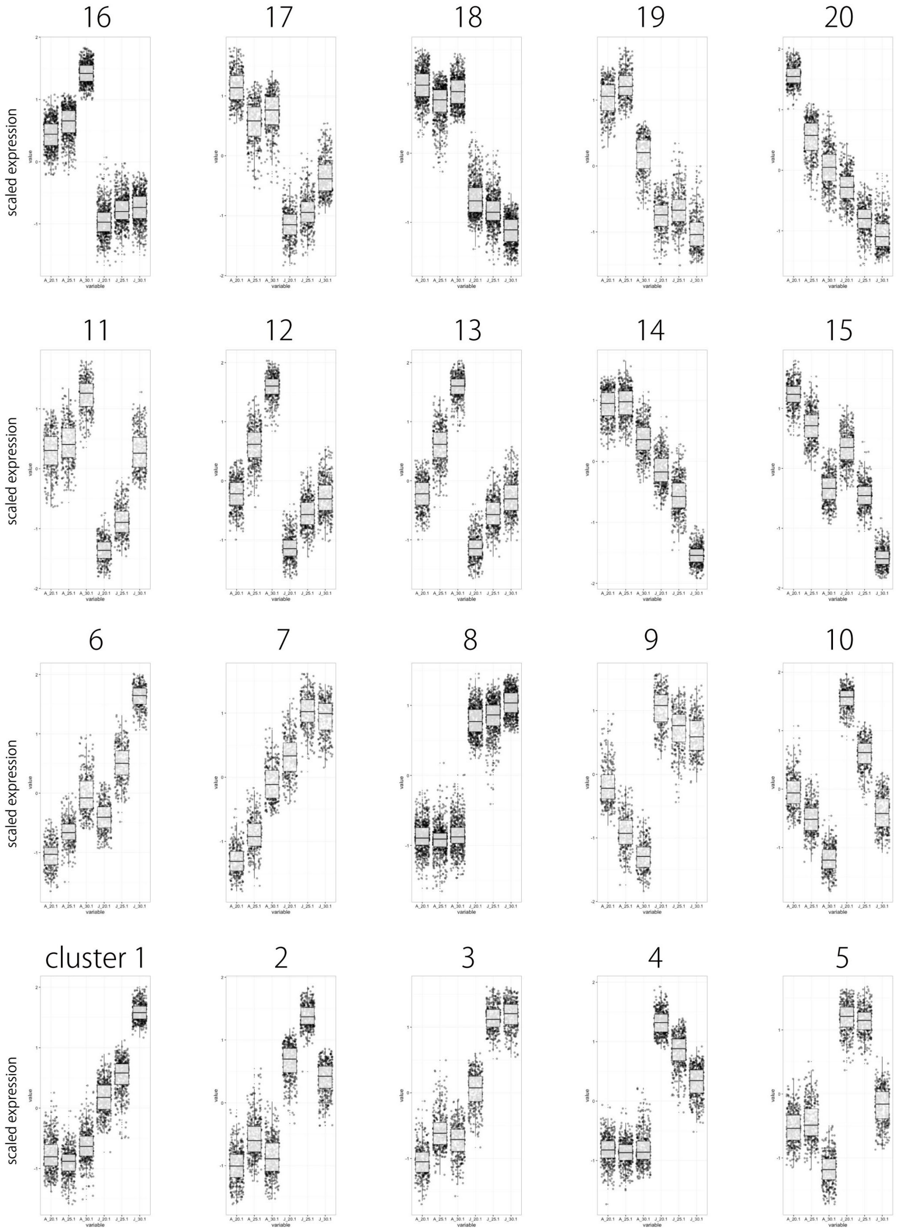

Supplementary Figure S3. Box plots showing expression patterns of each cluster. Scaled expression between accessions plotted during six different conditions (two accessions, three temperatures) is shown. Box plot explanation: upper horizontal line of box, 75th percentile; lower horizontal line of box, 25th percentile; horizontal bar within box, median; upper horizontal bar outside box, 90th percentile; lower horizontal bar outside box, 10th percentile.

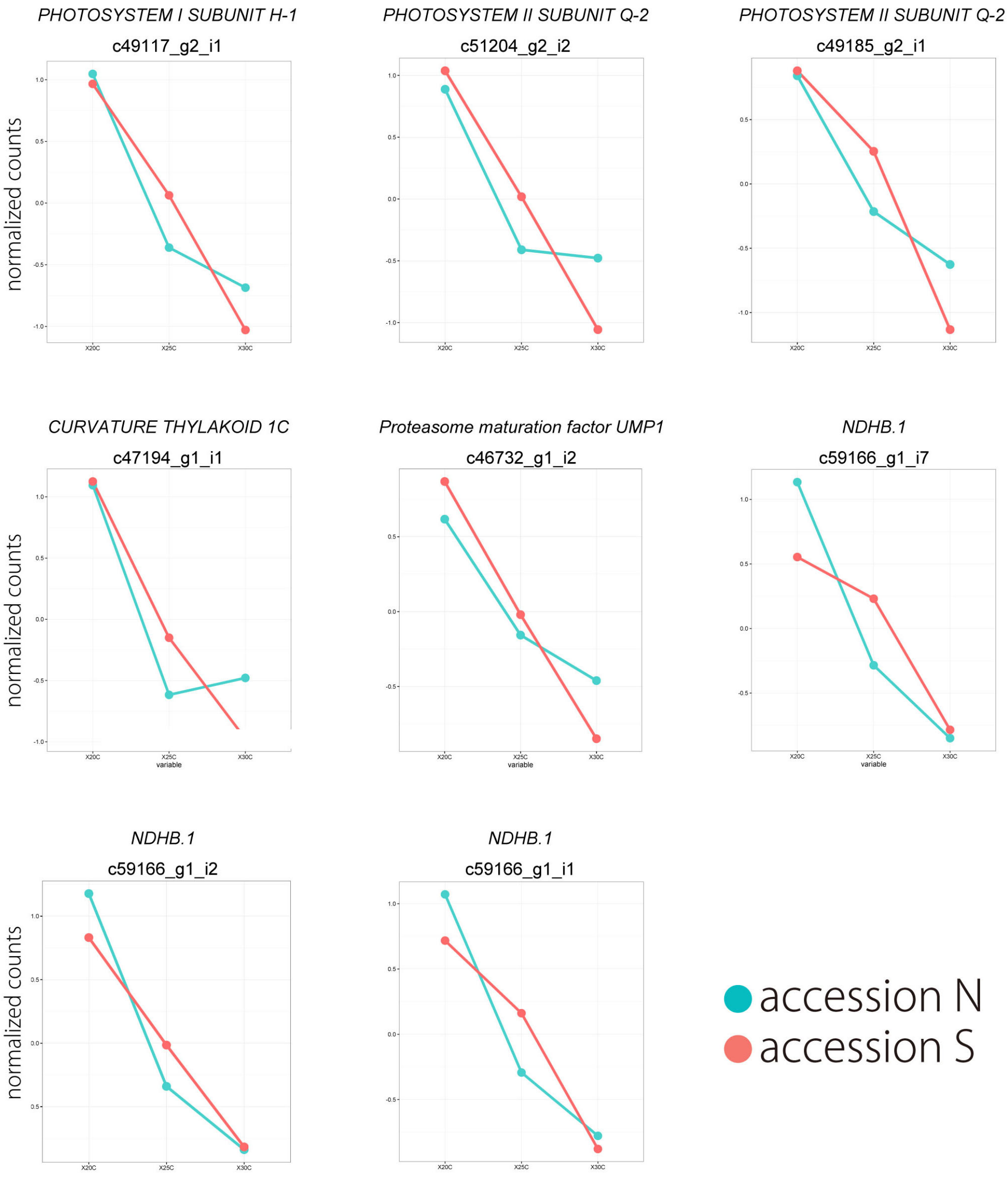

Supplementary Figure S4. Expression patterns of photosynthesis-related genes that had been displaced from cluster 9 to 6.

Normalized read counts for *PHOTOSYSTEM I SUBUNIT H-1*, *PHOTOSYSTEM II SUBUNIT Q-2*, *CURVATURE THYLAKOID 1C*, *UMP1*, and *NDHB.1*.

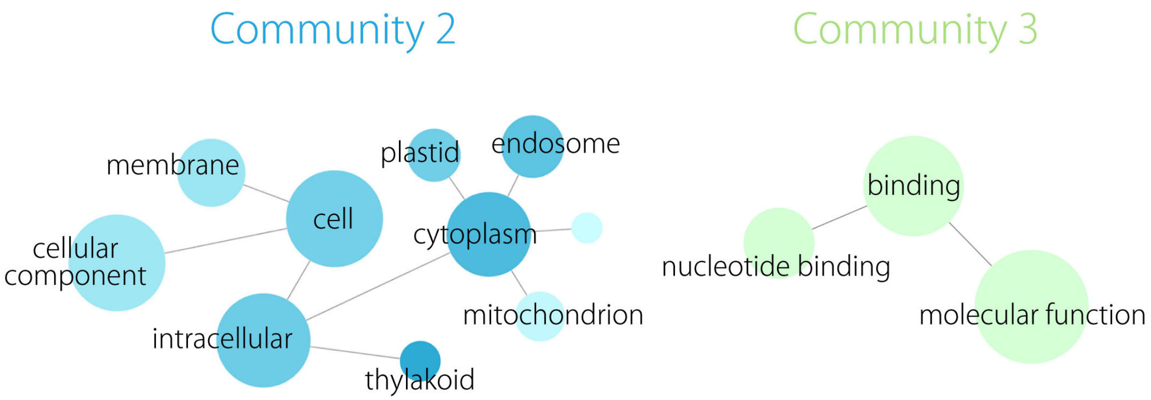

Supplementary Figure S5. GO enrichment map with differentially expressed genes that had been displaced from cluster 9 to cluster 6.

GO enrichment maps of communities 2 and 3 from Fig. 7A.

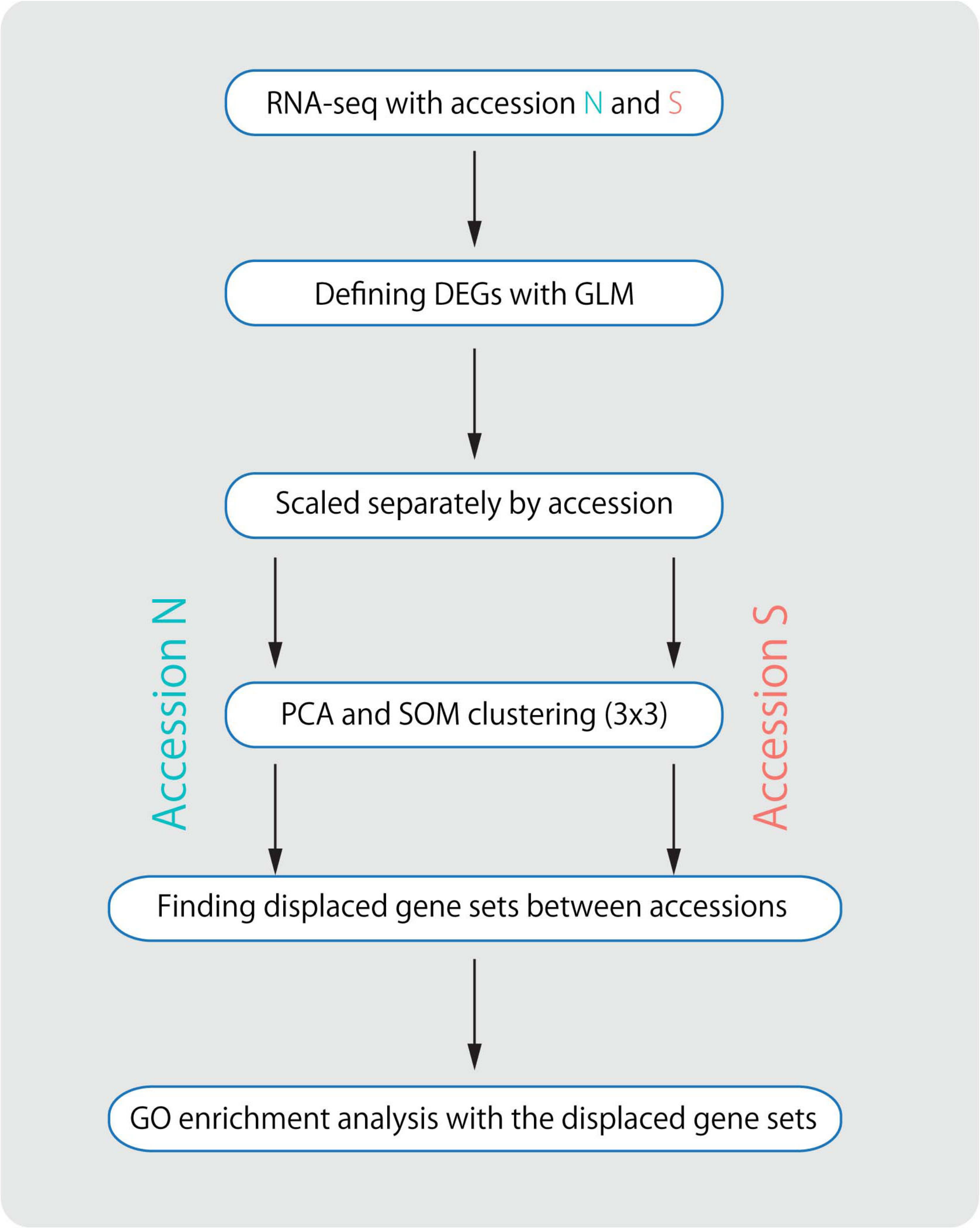

Supplementary Figure S6. Overview of RNA-seq and bioinfomatics to investigate differences between accessions.

Supplementary Table S2. Putative description of genes displaced from cluster 9 to cluster 6. Only genes, which we can find their putative ortholog in Arabidopsis, are indicated.

contig	GO	AGI code in Arabidopsis	description	p value
c51204_g2_i2	15980: photosynthesis	AT4G05180	photosystem II subnuit Q-2	1.58E-112
c47194_g1_i1	15983: photosynthesis	AT1G52220	CURVATURE THYLAKOID 1C	4.95E-60
c49185_g2_i1	15982: photosynthesis	AT4G05180	photosystem II subunit Q-2	1.58E-112
c46732_g1_i2	15979: photosynthesis	AT1G67250	Proteasome maturation factor UMP1	4.57E-68
c59166_g1_i7	15986: photosynthesis	ATCG00890	NAD(P)H-quinone oxidoreductase subunit 2 A	7.10E-145
c44774_g1_i1	15987: photosynthesis	AT1G76550	pyrophosphate--fructose-6-phosphate 1-phosphotransferase	3.10E-93
c59166_g1_i2	15988: photosynthesis	ATCG00890	NAD(P)H-quinone oxidoreductase subunit 2 A	8.16E-145
c49117_g2_i1	15989: photosynthesis	AT3G16140	photosystem I subunit H-1	2.65E-70
c59166_g1_i1	15990: photosynthesis	ATCG00890	NAD(P)H-quinone oxidoreductase subunit 2 A	7.28E-145
c58052_g3_i1	15984: photosynthesis	AT5G08050	uncharacterized protein	7.62E-70
c48657_g1_i1	15981: photosynthesis	AT5G27560	uncharacterized protein	7.37E-169
